# Supplementary material for: Social coding of the multisensory space around us
Source: R Soc Open Sci. 2019 Aug 7;6(8):181878. doi: 10.1098/rsos.181878 (PMC6731720; doi:10.1098/rsos.181878)
Supplement: Supplementary materials and results for Hobeika et al Social coding of the multisensory space around us [file rsos181878supp1.pdf]

1. RTs processing

The performances were analyzed in terms of RTs. Given that it is well known that the distribution of RTs is not normal [1,2], we used the natural logarithm transformation of RTs (ln) before trimming outlier RTs from the analyses. For each subject, each delay and each hemispace separately, we calculated the mean and the standard deviation of our transformed data. Ln(RTs) were considered outliers if they exceeded more than two standard deviations from the mean ln(RTs) and trimmed from the analyses (5.38% of trials for the audience experiment, 5.20% of trials for the collaborative experiment, 5.62% of trials in the competitive experiment). For the analyses, ln(RTs) were averaged for each subject and for each of the 44 conditions separately (2 HEMISPACE * 11 DISTANCE * 2 CONDITION). The means we obtained were transformed back with an exponential function.

2. Collaborative experiment

2.1 Outliers' participants

Among the participants, three participants had a high rate of misses in at least one of the social condition (isolated condition: 10% and 18.1% of miss, m±sd of the sample: 1.8 ± 3.8%, in a dyad condition: 14.5% of miss, m±sd of the sample: 1.2 ± 2.7 %) and were therefore excluded from the RTs analyses. The remaining participants were accurate in performing the task as the rates of omissions (0.70 ± 1.8%) can attest. We also excluded one participant from the analyses because his mean RT was substantially elevated (497.67 ± 127.76ms, m±sd of the sample: 364.55 ± 57.88ms), giving us reason to suspect that he did not correctly perform the task. There were twenty-six remaining subjects (14 females, age 24.08 ± 4.39).

2.2 Comparison between unimodal and bimodal trials

We first verified that multisensory integration globally speed up tactile detection in bimodal trials compared to unimodal trials. We conducted an ANOVA on the mean RTs, with the within subject factors DELAY (11 levels: Tactile_Before, T1, T2, T3, T4, T5, T6, T7, T8, T9 and Tactile_After). The global effect of DELAY was significant ($F_{(10,250)} = 61.23$, $p < .001$, $\eta_p^2 = .710$) suggesting that RTs were influenced by the time of tactile stimulation delivery (see figure S1 panel a).

RTs in the unimodal trials Tactile_Before were significantly slower than RTs in the bimodal trials T2, T3, T4, T5, T6, T7, T8 and T9 (Post-hoc Newman-Keuls' test: $p < .001$ in all cases, suggesting that sound presence boosted tactile detection at those delays. RTs at Tactile_Before did not significantly differ from RTs in the bimodal trials at T1 (Post-hoc Newman-Keuls' test: $p = .967$). RTs

in the bimodal trials at T1 were also significantly slower than all the other RTs in the bimodal trials (at T2, T3, T4, T5, T6, T7, T8 and T9; Post-hoc Newman-Keuls' test: p < .001 in all cases) suggesting that, even though the sound started at the same time as the tactile stimulus delivery in that condition, there was no multisensory integration. Thus, RTs at T1 were excluded from the rest of the analysis.

RTs in the unimodal trials Tactile_After were significantly faster than RTs at Tactile_Before (Post-hoc Newman-Keuls' test: p < .001). Given that RTs at Tactile_After were significantly slower than RTs at T7, T8 and T9 (Post-hoc Newman-Keuls' test: p < .001), we can exclude the possibility that participants were faster at late delays because of the increasing probability of receiving a tactile stimulation along trial.

2.3 Verification of the anisotropy of lateral PPS

We verified PPS anisotropy [3] by examining the effect of HEMISPACE on bimodal RTs. We conducted an ANOVA on the mean RTs with the within-subjects factors HEMISPACE (2 levels: Left/Right) and DELAY (8 levels: T2, T3, T4, T5, T6, T7, T8 and T9). The ANOVA revealed a significant main effect of DELAY ($F_{(7,175)}$ = 42.29, p < .001, η_p^2 =.629). The analysis also revealed that the two-way interaction HEMISPACE x DELAY was significant ($F_{(7,175)}$ = 5.17, p <.001, η_p^2 =.171), indicating that tactile detections were dependent on the temporal delay of tactile stimulation delivery from sound onset and the hemispace of origin of the sound source. The effect of the factor HEMISPACE was not significant. Thus, we analyzed the effect of the social manipulation with the other factors separately on the left and on the right PPS boundaries.

3. Competitive experiment

3.1 Outliers' participants

Among the participants, one had a high rate of misses (5.45% of miss, m±sd of the sample 1.24 ± 1.08%) and was therefore excluded from the analyses. The 29 remaining participants (16 females, age m±sd: 23.45 ± 4.15) were accurate in performing the task.

3.2 Comparison between unimodal and bimodal trials

We first verified that multisensory integration globally speed up tactile detection in bimodal trials compared to unimodal trials. We conducted an ANOVA on the mean RTs, with the within subject factors DELAY (11 levels: Tactile_Before, T1, T2, T3, T4, T5, T6, T7, T8, T9 and Tactile_After). The global effect of DELAY was significant ($F_{(10,280)}$ = 76.34, p <.001, η_p^2 = .732) suggesting that RTs

were influenced by the time of tactile stimulation delivery (see figure S1 panel b).

RTs in the unimodal trials Tactile_Before were significantly slower than RTs in the bimodal trials T2, T3, T4, T5, T6, T7, T8 and T9 (Post-hoc Newman-Keuls' test: p < .001 in all cases), suggesting that sound presence boosted tactile detection at those delays. RTs at Tactile_Before also significantly differed from RTs in the bimodal trials at T1 (Post-hoc Newman-Keuls' test: p = .011). RTs in the bimodal trials at T1 were also significantly slower than all the other RTs in the bimodal trials (at T2, T3, T4, T5, T6, T7, T8 and T9; Post-hoc Newman-Keuls' test: p < .001 in all). Thus T1 significantly differed from Tactile_Before, but also to all other bimodal trials. The expected behavioral effect of multisensory is an acceleration of RTs around 25ms at least (as seen in the other two experiments, and in the literature [4,5]). As Tactile_Before differs from T1 of 7ms and from T2 of 24ms, it is likely that the stimulation was not perceived as bimodal. Thus, T1 was excluded of the rest of the analysis.

RTs in the unimodal trials Tactile_After were significantly faster than RTs at Tactile_Before (Post-hoc Newman-Keuls' test: p < .001). Given that RTs at Tactile_After were significantly slower than RTs at T7, T8 and T9 (Post-hoc Newman-Keuls' test: p < .001), we can exclude the possibility that participants were faster at late delays because of the increasing probability of receiving a tactile stimulation along trial.

3.3 Verification of the anisotropy of lateral PPS

We verified PPS anisotropy [3] by examining the effect of HEMISPACE on bimodal RTs. We conducted an ANOVA on the mean RTs with the within-subjects factors HEMISPACE (2 levels: Left/Right) and DELAY (8 levels: T2, T3, T4, T5, T6, T7, T8 and T9). The ANOVA revealed a main significant effect of DELAY ($F_{(7,196)} = 44.27$, $p < .001$, $\eta_p^2 = .613$). The analysis also revealed that the two-way interaction HEMISPACE x DELAY was significant ($F_{(7,196)} = 2.90$, $p < .01$, $\eta_p^2 = .094$), indicating that tactile detections were dependent on the temporal delay of tactile stimulation delivery from sound onset and the hemispace of origin of the sound source. The effect of the factor HEMISPACE was not significant. Thus, we analyzed the effect of the social manipulation with the other factors separately on the left and on the right PPS boundaries.

4. Audience experiment

4.1 Outliers' participants

Among the 28 participants who took part to the Audience experiment, two had a high rate of misses in

at least one of the two social conditions (Isolated condition: 15.9% of miss, m±sd of the sample: 1.4 ± 3.1%; In Dyad condition: 17.3% and 28% of miss, m±sd of the sample: 2.7 ± 6.4 %) and were therefore excluded from the RTs analyses. The 26 remaining participants (11 females, age m±sd: 25.27 ± 3.58) were accurate in performing the task as the rates of omissions (0.87 ± 2.0%) can attest.

4.2 Comparison between unimodal and bimodal trials

We first verified that multisensory integration globally speed up tactile detection in bimodal trials compared to unimodal trials. We conducted an ANOVA on the mean RTs, with the within subject factors DELAY (11 levels: Tactile_Before, T1, T2, T3, T4, T5, T6, T7, T8, T9 and Tactile_After). The global effect of DELAY was significant ($F_{(10,250)} = 50.30$, $p < .001$, $\eta_p^2 = .668$) suggesting that RTs were influenced by the time of tactile stimulation delivery (see figure S1 panel c).

RTs in the unimodal trials Tactile_Before were significantly slower than RTs in the bimodal trials T2, T3, T4, T5, T6, T7, T8 and T9 (Post-hoc Newman-Keuls' test: $p < .001$ in all cases), suggesting that sound presence boosted tactile detection at those delays. RTs at Tactile_Before did not significantly differ from RTs in the bimodal trials at T1 (Post-hoc Newman-Keuls' test: $p = .260$). RTs in the bimodal trials at T1 were also significantly slower than all the other RTs in the bimodal trials (at T2, T3, T4, T5, T6, T7, T8 and T9; Post-hoc Newman-Keuls' test: $p < .001$ in all cases) suggesting that, even though the sound started at the same time as the tactile stimulus delivery in that condition, the auditory stimulus was not perceived by participants. Thus, RTs at T1 were excluded from the rest of the analysis.

RTs in the unimodal trials Tactile_After were significantly faster than RTs at Tactile_Before (Post-hoc Newman-Keuls' test: $p < .001$). Given that RTs at Tactile_After were significantly slower than RTs at T7, T8 and T9 (Post-hoc Newman-Keuls' test: $p < .02$), we can exclude the possibility that participants were faster at late delays because of the increasing probability of receiving a tactile stimulation along trial.

4.3 Verification of the anisotropy of lateral PPS

We verified PPS anisotropy [3] by examining the effect of HEMISPACE on bimodal RTs. We conducted an ANOVA on the mean RTs with the within-subjects factors HEMISPACE (2 levels: Left/Right) and DELAY (8 levels: T2, T3, T4, T5, T6, T7, T8 and T9). The ANOVA revealed a main significant effect of DELAY ($F_{(7,175)} = 35.99$, $p < .001$, $\eta_p^2 = .590$). The analysis also revealed that the two-way interaction HEMISPACE x DELAY was marginally significant ($F_{(7,175)} = 1.81$, $p = .08$, $\eta_p^2 = .067$), suggesting that tactile detections might be dependent on the temporal delay of tactile

stimulation delivery from sound onset and the hemispace of origin of the sound source. The effect of the factor HEMISPACE was not significant. Thus, according to the results of the two precedents experiences and of the literature, we analyzed the effect of the social manipulation with the other factors separately on the left and on the right PPS boundaries.

References

1. Luce RD. 1986 *Response times: Their role in inferring elementary mental organization (No. 8)*. Oxford Uni.
2. Ulrich R, Miller J. 1993 Information processing models generating lognormally distributed reaction times. *J. Math. Psychol.* **37(4)**, **513**.
3. Hobeika L, Viaud-Delmon I, Taffou M. 2018 Anisotropy of lateral peripersonal space is linked to handedness. *Exp. brain Res.* **236**, 609–618.
4. Canzoneri E, Magosso E, Serino A. 2012 Dynamic sounds capture the boundaries of peripersonal space representation in humans. *PLoS One* **7**, e44306. (doi:10.1371/journal.pone.0044306)
5. Taffou M, Viaud-Delmon I. 2014 Cynophobic Fear Adaptively Extends Peri-Personal Space. *Front. Psychiatry* **5**, 3–9. (doi:10.3389/fpsyt.2014.00122)

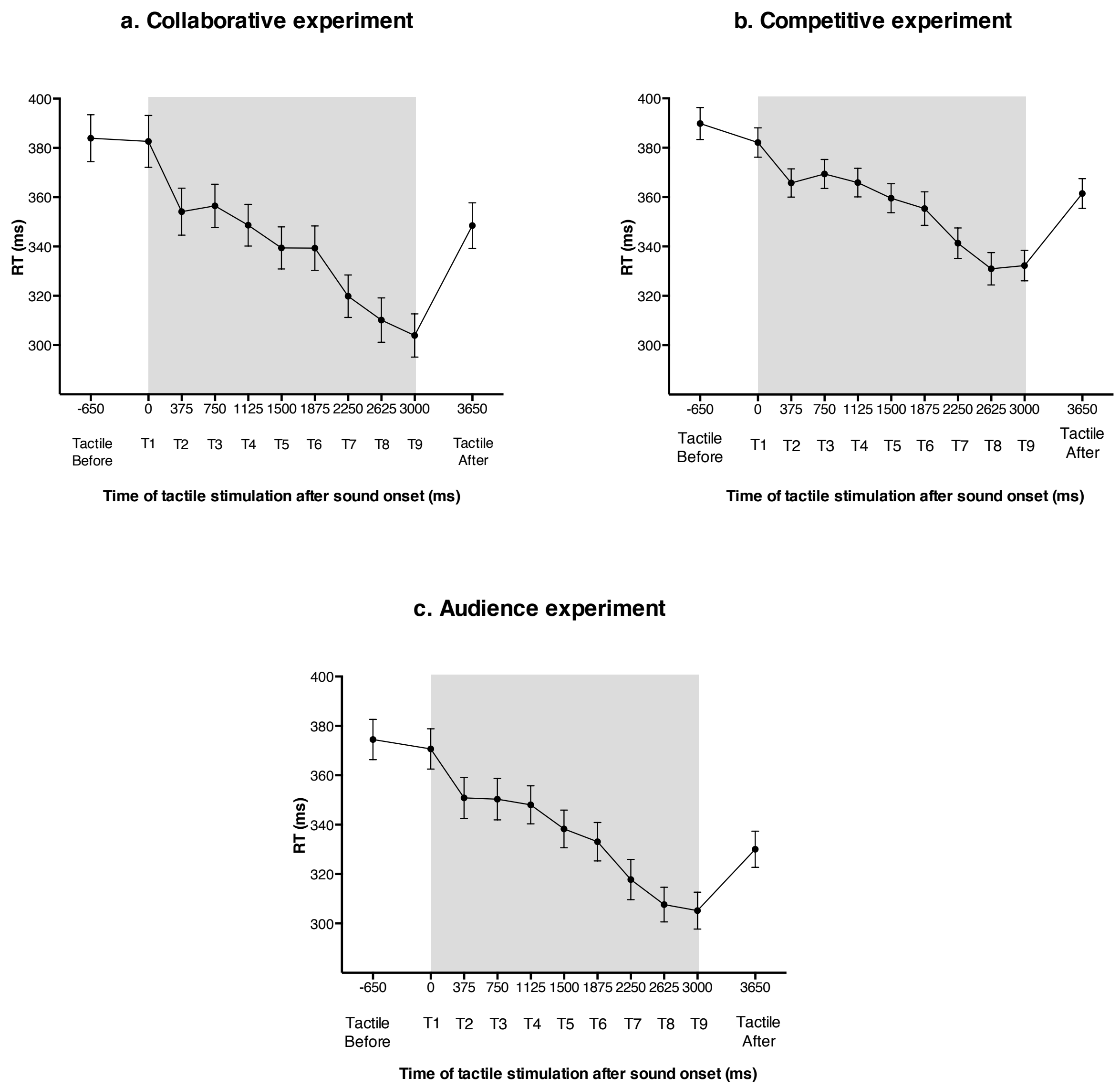

Figure S1. Impact of sound presence on tactile detection. Those figures reports participants' mean tactile reaction times (±SEM) as a function of the delay of tactile stimulation delivery from sound onset, on bimodal trials (from T1 corresponding at the sound onset, when the sound is the furthest from participants' body to T9 corresponding to the moment when the sound is the closest from participants' body) and on unimodal trials (Tactile_Before, Tactile_After) in the collaborative experiment (**panel a),** the competitive experiment (**panel b),** and the audience experiment (**panel c)**. The shaded region indicated the duration of the sound. Sound presence boosted tactile detection in bimodal trials, expect in T1 in which tactile stimulation occurred at sound onset.
